# Examining Genetic Variants Associated with FOXP1 Syndrome through Molecular Dynamics of Its DNA-Binding Domain and Self-Organizing Maps

**DOI:** 10.1021/acs.jcim.6c00080

**Published:** 2026-04-17

**Authors:** Stefano Motta, Nunzio Perta, Alice Romagnoli, Jesmina Rexha, Joseph D. Buxbaum, Silvia De Rubeis, Daniele Di Marino

**Affiliations:** † Department of Earth and Environmental Sciences, 9305 University of Milano-Bicocca, Milano, MI 20126, Italy; ‡ Inter-University Center for the Promotion of the 3Rs Principles in Teaching & Research (Centro 3R), Pisa 56122, Italy; § Department of Life and Environmental Sciences, 98854 Polytechnic University of Marche, Ancona, AN 60131, Italy; ∥ New York-Marche Structural Biology Center, Polytechnic University of Marche, Ancona, AN 60131, Italy; ⊥ Department of Neuroscience, Mario Negri Institute for Pharmacological Research-IRCCS, Milano, MI 20156, Italy; # Seaver Autism Center for Research and Treatment, 505555 Icahn School of Medicine at Mount Sinai, New York, New York 10029, United States; ∇ Department of Psychiatry, Icahn School of Medicine at Mount Sinai, New York, New York 10029, United States; ○ The Mindich Child Health and Development Institute, Icahn School of Medicine at Mount Sinai, New York, New York 10029, United States; ◆ Friedman Brain Institute, Icahn School of Medicine at Mount Sinai, New York, New York 10029, United States; ¶ Alper Center for Neural Development and Regeneration Friedman Brain Institute, Icahn School of Medicine at Mount Sinai, New York, New York 10029, United States

## Abstract

Genetic mutations in the transcription factor FOXP1 (forkhead box protein P1) cause an autosomal dominant neurodevelopmental disorder called FOXP1 syndrome. To understand the structural impact of pathogenic variants associated with FOXP1 syndrome, we investigated the conformational changes resulting from six distinct missense variants in FOXP1 by combining molecular dynamics simulations, molecular docking, and machine learning via self-organizing maps. Our results reveal different conformational landscapes mapped by the FOXP1 variants and reduced interactions with the DNA for mutations residing in helix H3 of the DNA-binding domain. These analyses offer a framework for assessing the structural impact of missense variants implicated in the FOXP1 syndrome, highlighting the importance of structural inferences in interpreting genetic variants.

## Graphical abstract

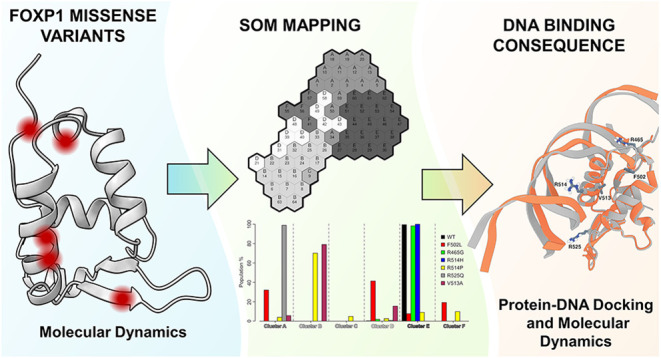

## Introduction

Interpreting the functional impact of missense genetic variants represents one of the most pressing and complex challenges in modern genomics and computational structural biology.^1^ Although sequencing technologies identify a growing number of variants associated with pathologies,^2,3^ deciphering the molecular mechanisms by which a single amino acid substitution alters protein function remains a significant hurdle. The perturbations induced by these mutations are often subtle, manifesting as changes in stability, conformational dynamics, or intermolecular interactions—effects that are difficult to capture with purely experimental approaches or static structural models.^4^ In this context, molecular dynamics (MD) simulations and advanced data analysis techniques, such as machine learning, are emerging as indispensable tools for providing an atomistic resolution of pathogenic mechanisms.^5,6^

An example of this challenge is FOXP1 syndrome, an autosomal dominant neurodevelopmental condition caused by pathogenic variants of the transcription factor FOXP1. This syndrome is characterized by early motor and language delays, intellectual disability (ID), speech and language deficits, behavioral problems including autism spectrum disorder (ASD), and additional comorbidities (MIM #613670).^7–15^ Pathogenic *FOXP1* variants are typically *de novo* and can be protein-truncating variants leading to haploinsufficiency or missense variants.^9,11,13–16^ To date, at least 200 cases of FOXP1 syndrome have been reported.^8^ However, no clear genotype-phenotype correlations have been established, even when considering recurrent variants.^7,16,17^

FOXP1, FOXP2, FOXP3, and FOXP4 form the FOXP family of transcription factors, characterized by a conserved winged-helix/forkhead DNA-binding domain.^18^ All of the FOXP family members are linked to genetic conditions. Heterozygous variants in *FOXP2* can cause an autosomal dominant speech and language disorder (MIM #602081).^19,20^ *FOXP4* haploinsufficiency is associated with an autosomal dominant neurodevelopmental condition featuring speech and language deficits, delayed growth, and variable congenital abnormalities.^21,22^ Pathogenic variants in *FOXP3* are associated with X-linked immunodysregulation, polyendocrinopathy, and enteropathy (MIM #304790).^23^

FOXP1 is a fundamental regulator of gene expression in neural tissues^24–27^ and studies using preclinical experimental models have demonstrated its essential role in the development of the neocortex^28–30^ and the striatum.^25,27,31^ FOXP1 operates in concert with other transcription factors implicated in neurodevelopmental disorders,^24,32^ including FOXP2.^33^ Structurally, FOXP1 includes a C-terminal forkhead (FKH) DNA-binding domain and three regulatory regions involved in protein–protein interactions: an N-terminal glutamine-rich region (Q-rich), a zinc finger, and a leucine zipper (ZIP). The FKH domain consists of three α-helices (i.e., H1, H2, and H3), three β-strands (i.e., S1, S2, and S3), and two wings (i.e., W1 and W2).^34^ This domain typically exists as a monomer, with the helix H3 contacting the major groove of DNA.^35^ An ∼90-residue flexible linker separates the FKH from the ZIP domains, enabling these structural elements to facilitate the formation of homo- and heterodimers, including with FOXP2, via a mechanism known as domain swapping.^36,37^ Domain swapping involves two or more proteins exchanging identical structural regions, a process that often requires the unfolding of the monomer.^38^ In FOXP1, however, the existence of a native-like monomeric intermediate in the folding pathway of the three-dimensional domain-swapper dimer indicates that dimer formation occurs without complete monomer unfolding.^39^ Interestingly, opposed to the conventional folding-upon-binding mechanism, DNA stabilizes the monomeric form of FOXP1 while destabilizing the three-dimensional domain-swapped dimer.^33,40^ These structural changes promote the accumulation of a disordered ensemble and shift the dimerization equilibrium toward the monomeric state. The DNA-bound monomer thus acts as a negative allosteric regulator of dimerization by increasing the energy barrier for dimer formation.^40^ Importantly, while FOXP proteins exhibit similar dynamics in their monomeric form, they differ significantly in their behavior when dimerized.^39^

To the best of our knowledge, no *in silico* studies of pathogenic missense variants in FOXP1 have been conducted. In this study, we developed and applied an integrated computational approach that combines large-scale MD simulations, an unsupervised machine learning technique—self-organizing maps (SOMs)—and molecular docking. The extensive use of MD replicas allowed us to robustly sample the energetic and dynamic landscape of the FOXP1 FKH domain, in both its wild-type form and in six syndrome-associated variants. To navigate the enormous amount of data generated, we employed SOMs, a class of artificial neural networks that project high-dimensional data onto a low-dimensional grid of ″neurons″, effectively creating a map of the conformational landscape explored during simulations. This approach is particularly powerful for analyzing the vast data sets generated by MD, as it preserves the topological relationships between sampled conformations, ensuring that similar structures are mapped to adjacent neurons.

This approach enabled us to identify and classify distinct conformational ″macrostates″, revealing how different mutations alter the domain’s dynamic behavior in specific and nonintuitive ways. Finally, to connect these structural changes to DNA-binding competence, we employed protein–DNA docking to assess how the different macrostates identified by the SOMs affected the domain’s ability to interact with its target. Our work not only provides new, detailed insights into the mechanisms of pathogenic FOXP1 variants but also demonstrates the power of a framework that integrates simulation and machine learning to decipher the impact of genetic variants. This approach offers a paradigm that can be applied to other protein systems, where the relationship between a point mutation and its functional consequences remains elusive.

## Materials and Methods

### Prioritization of FOXP1 Missense Genetic Variants for the Modeling

As reported by Trelles et al.,^10^ six *de novo* missense variants were identified within the forkhead-binding domain in patients clinically diagnosed with FOXP1 syndrome; thus in our study we tested: NM_001349338.3:c.1393A > G (p.Arg465Gly) denoted here as R465G, classified as pathogenic/likely pathogenic in ClinVar (#217264), and previously reported;^9,10,15^ c.1541G > A (p.Arg514His) denoted here as R514H, classified as pathogenic/likely pathogenic in ClinVar (#211040), and previously reported;^10,41^ c.1541G > C (p.Arg514Pro), denoted here as R514P, classified as likely pathogenic in ClinVar (#975881), and previously reported in^10,41^ c.1574G > A (p.Arg525Gln), denoted here as R525Q, classified as pathogenic/likely pathogenic in ClinVar (#521111), and previously reported in^10,42,43^ c.1506C > G (p.Phe502Leu), denoted here as F502L, classified as pathogenic/likely pathogenic in ClinVar (#391588), and previously reported;^9,10^ and, c.1538T > C (p.Val513Ala), denoted here as V513A, classified with conflicting interpretation as pathogenic/likely pathogenic in ClinVar (#522138), and previously reported.^10^

### Structures for Modeling

The wild-type (WT) system was developed starting from the NMR solution structure (PDB ID: 2KIU) of the FOXP1 DNA-binding domain.^34^ Residues mutated in the experimental structure were replaced with the corresponding native amino acids from the human sequence. The six models containing the missense variants were built using PyMOL (Schrödinger LLC, The PyMOL Molecular Graphics System, New York, 2010), mutating functionality and choosing the option with the fewest unfavorable contacts. The systems were then prepared using the Schrödinger Preparation Wizard tool.^44^ The preprocessing steps included the addition of hydrogen atoms, the removal of all water molecules, and the determination of residue protonation states using PROPKA. Each system was solvated with TIP3P water molecules in a cubic box with dimensions of approximately 7.6 × 7.6 × 7.6 nm ensuring a minimum distance from the box edges sufficient to avoid periodic boundary condition artifacts (minimal distance of the box boundaries from the solute of 14 Å). The final size of each simulated system comprised approximately 40000 total atoms, with minor variations depending on the specific mutation. Neutralizing counterions were added to reach a physiological salt concentration employing the GROMACS preparation tools.^45^ We used neutralizing counterions only to keep the ionic strength constant and minimal across systems and to emphasize mutation-dependent differences in native-state dynamics under a controlled electrostatic baseline. The simulations were performed using GROMACS 2018.1 ^46^ with the Amber14sb force field.^47^

### Molecular Dynamics (MD)

The WT and six mutant models underwent a series of equilibration steps. Initially, a steepest descent energy minimization was conducted, involving 2000 steps, with positional restraints (set at 2000 kJ mol^–1^ nm^–2^) applied exclusively to the backbone atoms. Subsequently, a 250 ps NVT MD simulation was executed to gradually raise the system’s temperature from 0 to 100 K, concurrently reducing the restraints to 500 kJ mol^–1^ nm^–2^. The system was then further heated to 300 K over a 500 ps NPT simulation, progressively relaxing the restraints to 250 kJ mol^–1^ nm^–2^. Following this, a 3 ns equilibration phase took place in an NPT simulation, with the gradual reduction of backbone restraints to 150 kJ mol^–1^ nm^–2^. Subsequently, a second 10 ns equilibration phase commenced, during which the restraints were reduced to 50 kJ mol^–1^ and nm^–2^. For each system, including the WT and the six variants, we performed 10 independent production replicas, each lasting 200 ns, for a total of 2 μs per system. Each replica was initialized independently by starting with different initial velocities. Throughout the subsequent 10 × 200 ns production runs, conducted in the isothermal–isobaric (NPT) ensemble at 300 K, all restraints were completely removed. During the NVT simulations, temperature regulation was achieved using the Berendsen thermostat^48^ with a coupling constant of 0.2 ps. In the NPT simulations, a V-rescale^49^ thermostat with a coupling constant of 0.1 ps was employed for temperature control, while pressure was maintained at 1 bar using a Parrinello–Rahman barostat^50^ with a coupling constant of 2 ps. The simulations employed a time step of 2.0 fs and included the application of the LINCS^51^ algorithm to constrain bonds involving hydrogen atoms. To manage long-range electrostatic interactions, the particle mesh Ewald method^52^ was utilized, with cutoff distances established at 12 Å. A similar equilibration protocol was applied in other cases.^53,54^ Trajectory analyses were performed using the GROMACS analysis tools.^46^ The cutoff distance for the number of contacts analysis was set to 4.5 Å.

The secondary structure was assigned along the trajectories by using *gmx dssp* (via the GROMACS tool). H1 was included as an internal control and remained consistently α-helical across WT and all variants with no cluster-dependent differences (Supporting Figures 1 and 2). Therefore, subsequent analysis focused on helices showing variant-dependent changes (H2–H4).

### Self-Organizing Maps (SOMs)

We used PathDetect-SOM^55,56^ to train an 8 × 8 toroidal SOM, which exhibits periodicity across its boundaries and adopts a hexagonal lattice structure. A toroidal architecture was chosen, as it is particularly well-suited for analyzing conformational sampling around various equilibrium states, as in this work. We selected an 8 × 8 grid size because our previous benchmarking on similar systems showed it provides an optimal descriptive resolution to identify relevant conformational macrostates without overpartitioning the structural landscape into functionally redundant microstates (which would be strictly necessary only for detailed kinetic network analyses^57^). Unlike nonperiodic maps that are ideal for visualizing processes with defined start and end points,^58^ a periodic SOM removes edge artifacts by ensuring all neurons have the same number of neighbors, thus providing a more homogeneous and continuous representation of the conformational landscape.^59,60^ The input features used for training consist of conformational snapshots extracted from the MD ensemble, mathematically encoded as a set of interatomic distances between Cβ atoms. Cβ atoms were selected as they optimally capture both the backbone topology and the initial side-chain orientation while minimizing the thermal noise generated by highly flexible surface side chains. Only the distances between atoms forming a contact (distance lower than 1.1 nm) in the experimental structure were considered, as in a previous work,^58^ resulting in a total of 765 pairwise distances. The 1.1 nm threshold was chosen based on the literature determining the optimal distance thresholds for reproducing protein contact maps and capturing native-state topologies.^61^ In the case of glycine residues, the Cα atom was considered instead of the Cβ atom. These distances were computed at regular intervals of 100 ps from the whole set of WT and mutant MD replicas. Subsequently, we conducted an agglomerative hierarchical clustering of the neurons using Euclidean distance and complete linkage, which resulted in six as the optimal number of clusters, determined by evaluating Silhouette profiles (Supporting Figure 3). Notably, given that the SOM employed here is toroidal, interpreting the visual output can be challenging. To address this challenge, we applied a visualization optimization protocol, as in previous studies.^59,62^ The DockQ scores were mapped onto the SOM by averaging the top five scores obtained by the 200 poses generated for each neuron (Figure 1). All analyses were conducted using the Kohonen package within the R statistical environment.^63,64^

**Figure 1:**
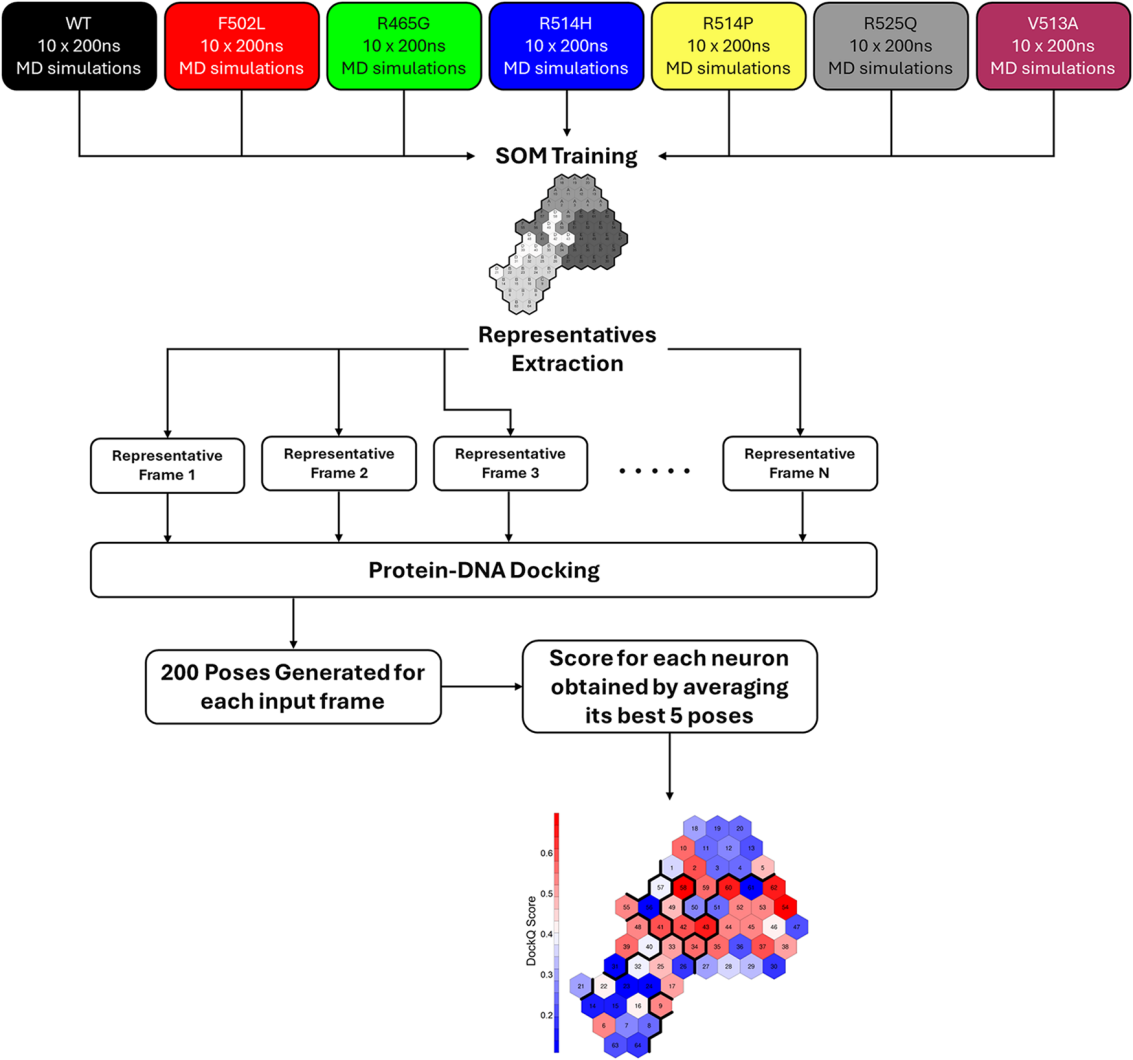
Scheme representing the protocol followed for MD simulation analysis, selection of representatives, protein–DNA docking, and final mapping of scores on the SOM.

### Protein–DNA Molecular Docking

The initial structures for WT and mutant FOXP1-DNA docking were obtained from representative structures extracted from SOM analysis. To ensure an exhaustive sampling of the conformational landscape, rather than limiting the analysis to the macrocluster representatives, we performed docking on the centroid of each individual SOM neuron. Specifically, for each of the 64 neurons, we extracted the individual MD frames that minimized the structural distance to the geometric center of that neuron. The 3D structure of the dsDNA was retrieved from the FOXP2-DNA complex with PDB ID 2A07,^65^ since the DNA sequences bound by these transcription factors are similar.^34,65^ Notably, the DNA binding residues within the FKH domain of FOXP2 and FOXP1 are conserved.^34,65,66^ The same DNA duplex has also been employed to experimentally quantify FOXP1-DNA binding (PMID: 21416545), reinforcing its validity as an FOXP forkhead-binding substrate and aligning with the conserved DNA-contacting determinants found across FOXP proteins. The dsDNA sequence used for all docking calculations was taken directly from the FOXP2-DNA complex (PDB ID: 2A07): strands 1-21, 5′-AACTATGAAACAAATTTTCCT-3′; strand 22–42, 5′-TTAGGAAAATTTGTTTCATAG-3′. The same dsDNA sequence was used for the WT and all FOXP1 variants to ensure comparability across the docking runs.

Docking was performed using the HADDOCK3 software, a modular integrative modeling tool tailored for protein–DNA docking (HADDOCK3, Bonvin’s Lab, https://github.com/haddocking/haddock3).^67^ Our workflow involved the following modules: (i) *topoaa*: generates all-atom topologies for the CNS engine; (ii) *rigid-body*: performs rigid-body energy minimization and samples 1000 structures; (iii) *caprieval* (analysis): calculates CAPRI metrics (i.e., i-RMSD, l-RMSD, Fnat, DockQ) relative to the reference structure (PDB ID: 2A07, chains A, B, and J); (iv) *seletop*: selects the top 200 models from the previous step; (v) *caprieval* (analysis); (vi) *flexref* (refinement): executes semiflexible refinement using a simulated annealing protocol via torsion angle space molecular dynamics simulation; (vii) *caprieval* (analysis); (viii) *mdref* (refinement): performs short explicit solvent Cartesian MD refinement, which consists of brief heating, MD at 300 K, and cooling. Using the default *mdref* settings, this corresponds to 100 MD steps for heating, 1250 MD steps at 300 K, and 500 MD steps for cooling (plus energy minimizations); (ix) *caprieval* (analysis). Our CAPRI evaluation utilized the monomeric FOXP2 bound to the DNA complex (PDB ID: 2A07, chains A, B, and J) as the reference structure. All further analyses were conducted based on the results obtained from step (viii) *mdref* and (ix) *caprieval*. In our study, we modified the electrostatic treatment for DNA by setting the dielectric parameter (″*dielec*″) to ″cdie″ with epsilon (ε) equal to 78. Additionally, we enabled automatic definition of DNA restraints to maintain the double helix structure by setting the ″*dnarest_on*″ parameter to ″true″. To generate restraints for the active residues involved in the interaction between FOXP1 and DNA, we used the GenTBL Web server. The selected active residues to drive the docking were N511, R514, H515, S518, L519, and W534 for FOXP1, and A12, A13, A14, T15, A30, T31, T32, and T33 for the dsDNA (Supporting Figure 4B). These same selected active residues were used consistently across all docking runs. For each neuron, all the 200 FOXP1-DNA complex structures obtained for the *mdref* step were individually inspected using PyMOL (Schrödinger LLC. The PyMOL Molecular Graphics System. New York 2010). The binding poses can be classified into four main conformational groups: resembling the monomeric form observed in the crystal complex of FOXP2-DNA (PDB ID: 2A07, chains A, B, and J), resembling the major groove binding mode seen in the dimeric switch form of the FOXP2-DNA crystal structure (PDB ID: 2A07, chains A, B, and J), binding to the minor groove, and outliers (refer to Supporting Figure 4).

To validate the docking poses, explicit solvent MD simulations (5 × 250 ns) were performed for the WT, F502L, and R514P protein–DNA complexes. The simulations were carried out using the same environmental parameters described above, employing the amber ff14SB force field for the protein and the parmbsc1 force field for the DNA. Interface native contacts (using a 4.0 Å distance cutoff between heavy atoms) and protein backbone RMSD (following structural alignment on the DNA phosphorus atoms) were calculated using cpptraj.

## Results

### The DNA-Binding Domain of FOXP1 Carrying Patient-Specific Mutations Shows Altered Local Flexibility

We performed MD simulations^68–71^ investigating six different *de novo* missense FOXP1 variants, each mapped onto the FKH DNA-binding domain and classified as likely pathogenic or pathogenic. Specifically, we simulated six separate systems, each carrying one of the following single-point mutations: R465G, F502L, V513A, R514H, R514P, and R525Q (see the Materials and Methods section for further details) (Figure 2a). We also included V513A, which was classified with conflicting interpretation (see the Materials and Methods section for further details) (Figure 2a).

**Figure 2:**
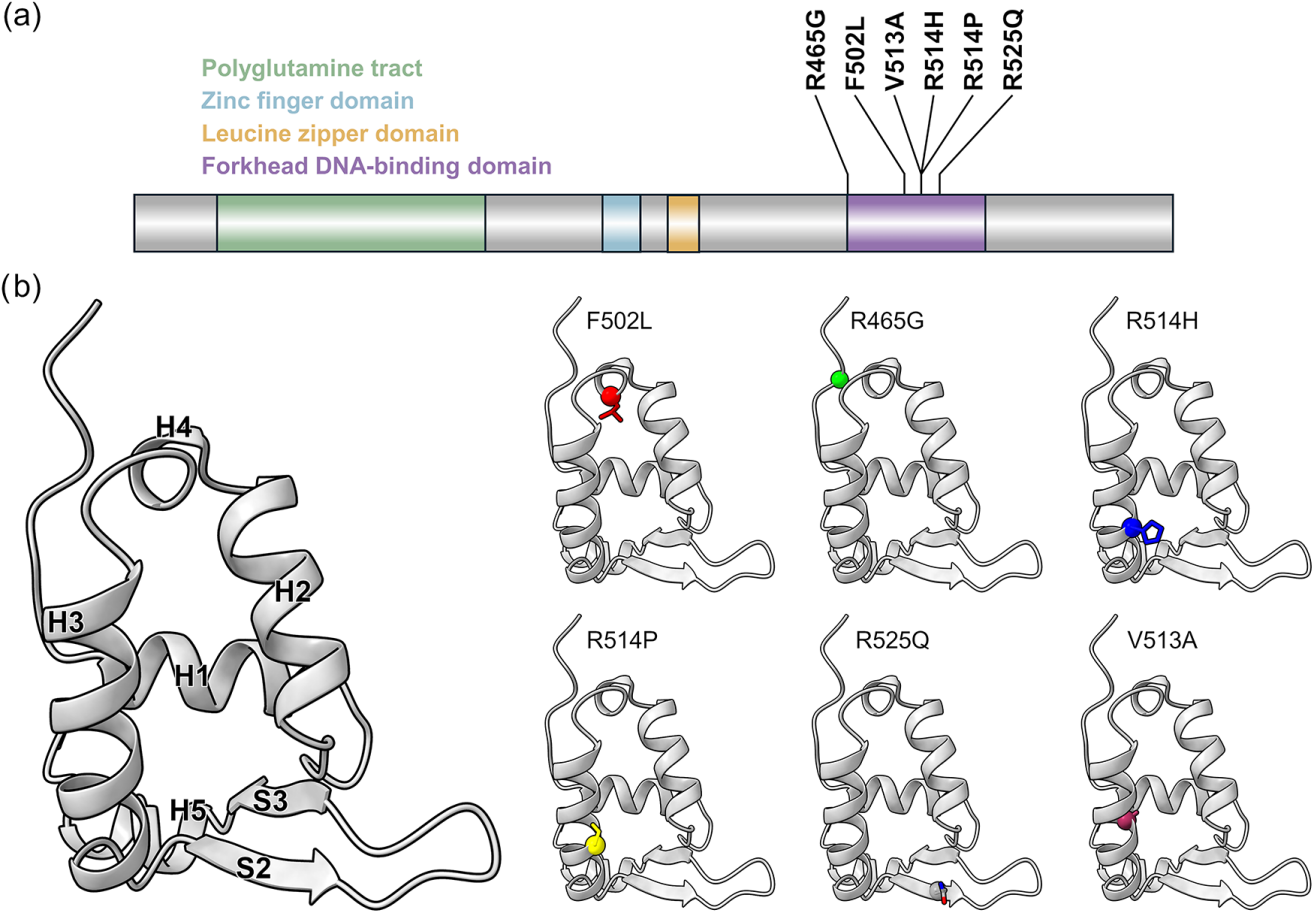
Localization of the six missense mutations analyzed in this study. (a) Topology of FOXP1, with the six missense mutations annotated. (b) Structure of the three-dimensional fold of the FOXP1 FKH DNA-binding domain, with annotated secondary structure elements (left panel) and mutations analyzed in this work (right panel).

As previously described,^34^ the FKH DNA-binding domain of FOXP1 exhibits a well-defined fold characterized by a four-helix bundle packed with a triple-stranded antiparallel β-sheet (Figure 2b). This structural arrangement is a hallmark of many DNA-binding domains found in transcription factors.^65^ The DNA binding is expected to be primarily mediated by the H3 helix, as observed in the FOXP2 structure.^65^ Three-dimensional domain swapping evidenced that the extension of the H2 helix through the turn that connects to the H3 helix is responsible for homodimerization.^65^ F502, V513, R514, R514, and R525 are in the H2–H3 region, and R465 is at the N-terminal portion of the domain. We performed 10 replicas of 200-ns-long MD simulations for the WT and mutant systems. Using such a high number of replicas allowed us to obtain reliable statistics over the differences captured during the MD simulations.

First, we assessed the overall structural deviations of the WT and mutant FOXP1 FKH DNA-binding domains by calculating the Root Mean Square Deviation (RMSD) of the backbone atoms over the course of the MD simulations (Figure 3). Based on the RMSD values, mutants R465G, V513A, R514H, R514P, and R525Q display a behavior comparable to the WT for most of the replicas. This was also confirmed by the small deviation in the total number of internal contacts of the domain monitored during the simulations (Supporting Figure 5). The F502L variant, on the other hand, showed RMSD values >1.5 Å in almost all replicas, indicating that this amino acid substitution has a significant impact on the structural deviation of the protein domain from the reference (Figure 3).

**Figure 3:**
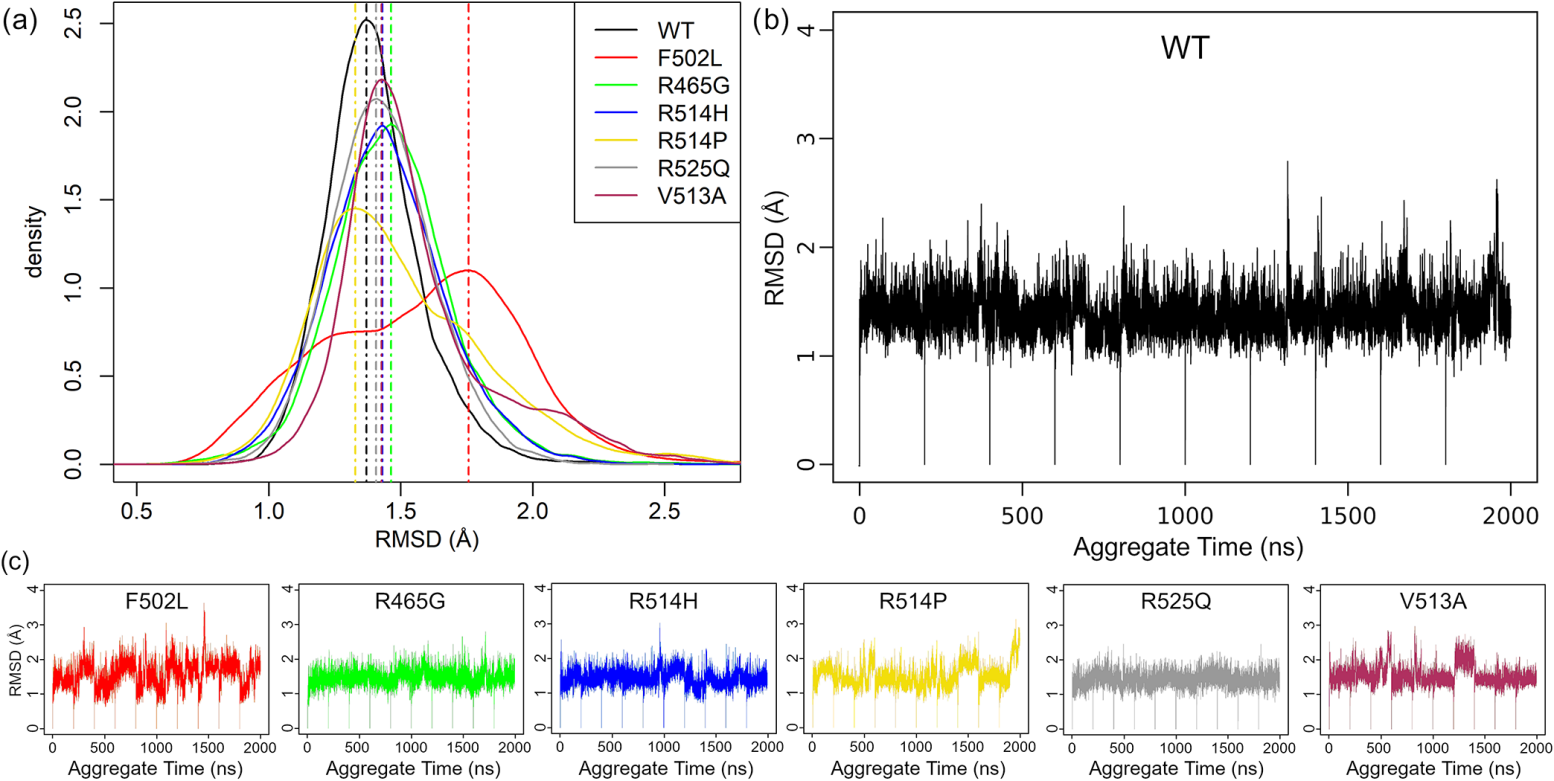
FOXP1 mutants show a higher degree of fluctuations compared to the WT, based on RMSD analysis. (a) Distribution of RMSD values for conformations sampled during MD simulations. (b) Monitoring of RMSD during concatenated replicas for the WT system. (c) Monitoring of RMSD during concatenated replicas for the mutated systems.

To further dissect local fluctuations and obtain residue-level insights into flexibility and local mobility, we conducted Root Mean Square Fluctuation (RMSF) analysis for the WT and FOXP1 mutants. We observed a high degree of fluctuations in the H2–H3 region (residues 489–517), where specific residues show elevated RMSF values (Figure 4). This fluctuation is higher for most mutants compared to that of the WT protein.

**Figure 4:**
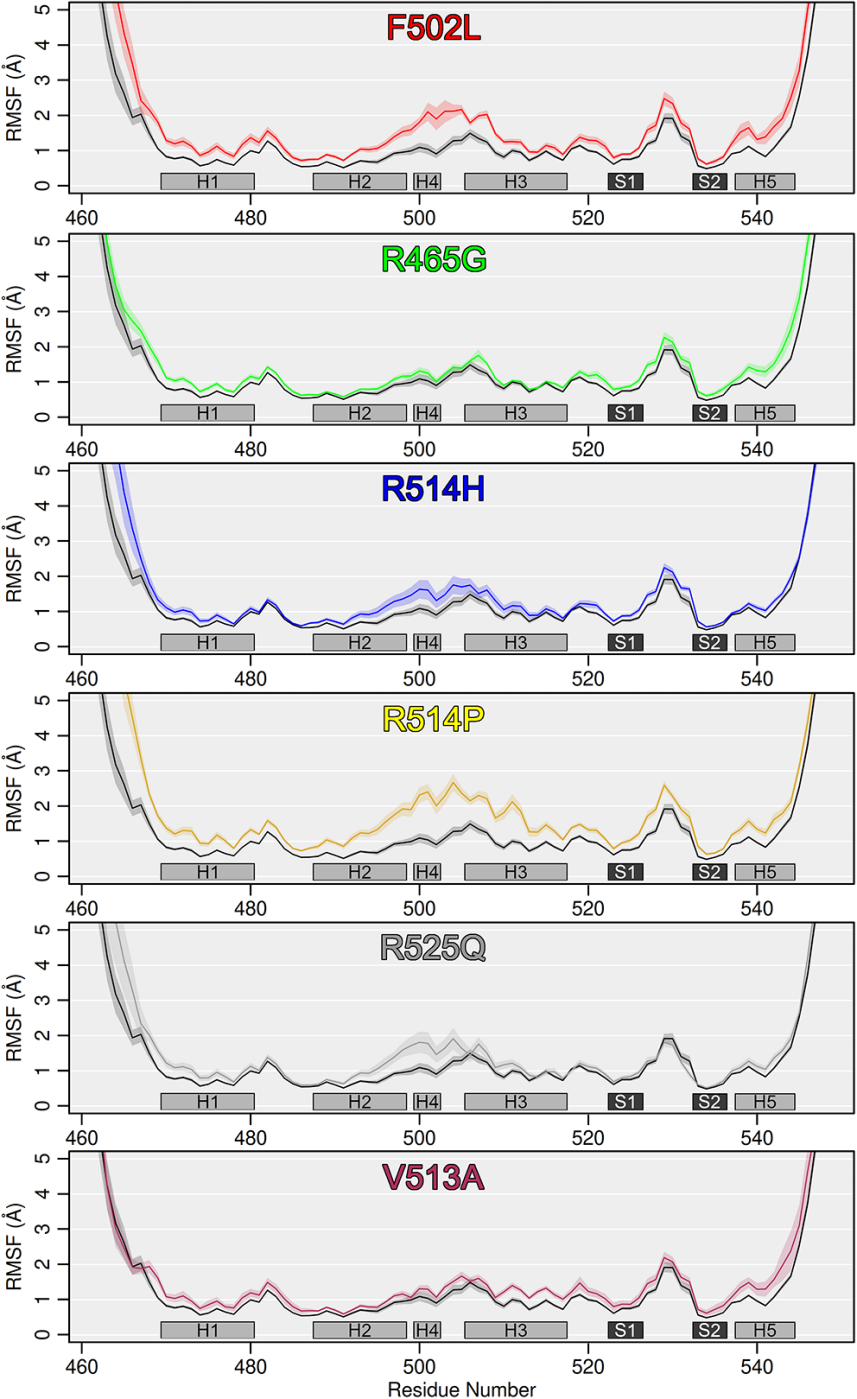
FOXP1 mutants show a high degree of local fluctuations, based on RMSF analysis. The solid lines represent the mean RMSF values computed across 10 independent 200 ns MD replicas for the FOXP1 mutants (colored lines) compared to the WT (black line) across the entire protein length. The shaded regions surrounding the lines indicate the standard error of the mean (SEM) per residue. Secondary structure elements are annotated at the bottom of each panel with light gray for helices and dark gray for sheets.

Overall, these analyses show that patient-specific missense variants associated with FOXP1 syndrome affect the dynamic behavior of the DNA-binding domain, potentially affecting the ability to dimerize or form stable interactions with the DNA.

### Self-Organizing Maps Identified Different Structural Behavior in FOXP1 FKH Domain

To further expand our understanding of the dynamics displayed by the FOXP1 mutants, we employed self-Organizing Maps (SOMs), a class of unsupervised artificial neural networks in which computational units, often referred to as “*neurons”*, are arranged in a grid-like structure, preserving topological relationships.^72^ These computational *neurons* do not correspond to biological neurons but are instead abstract units that adjust their weights during training to represent patterns in the data. SOMs transform multidimensional input data into a concise, low-dimensional representation where each unit (or neuron) corresponds to a feature vector with the same dimensions as the input data vectors. The training process of the SOM involves an iterative procedure in which neurons adjust to the input data, constructing a map that encapsulates the essential aspects of the input data set. SOMs are a type of artificial neural network, that have proven valuable in the analysis of various biomolecular processes ranging from protein–protein interactions,^59^ interactions with ligands,^60,62,73–75^ and protein folding.^58,76^

We trained an 8 × 8 toroidal SOM (with periodicity across the boundaries) using a set of interatomic distances between the Cβ atoms of the protein. The input features used for training consist of individual conformational snapshots extracted from the concatenated MD ensemble. Each snapshot was mathematically encoded as a high-dimensional vector of instantaneous interatomic distances between Cβ atoms (using Cα for glycine residues). To define the precise feature set, we selected all pairs forming a native contact (distance <1.1 nm) in the experimental WT reference structure, resulting in a total of 765 pairwise distances. These distances were computed at regular 100 ps intervals across all replicas to explicitly capture the dynamic variance of the system without time-averaging. During the training phase, SOM undergoes a process of optimization to effectively capture input conformations. Each input conformation is ultimately associated with a specific neuron represented as a hexagon on the SOM. Consequently, every neuron within the SOM serves as a representation of a distinct conformational microstate within the system, with neighboring neurons indicating similarity in configurations. These neurons are subsequently organized into compact clusters, each of which provides a succinct representation of the macrostates of the system (Figure 5a).

**Figure 5:**
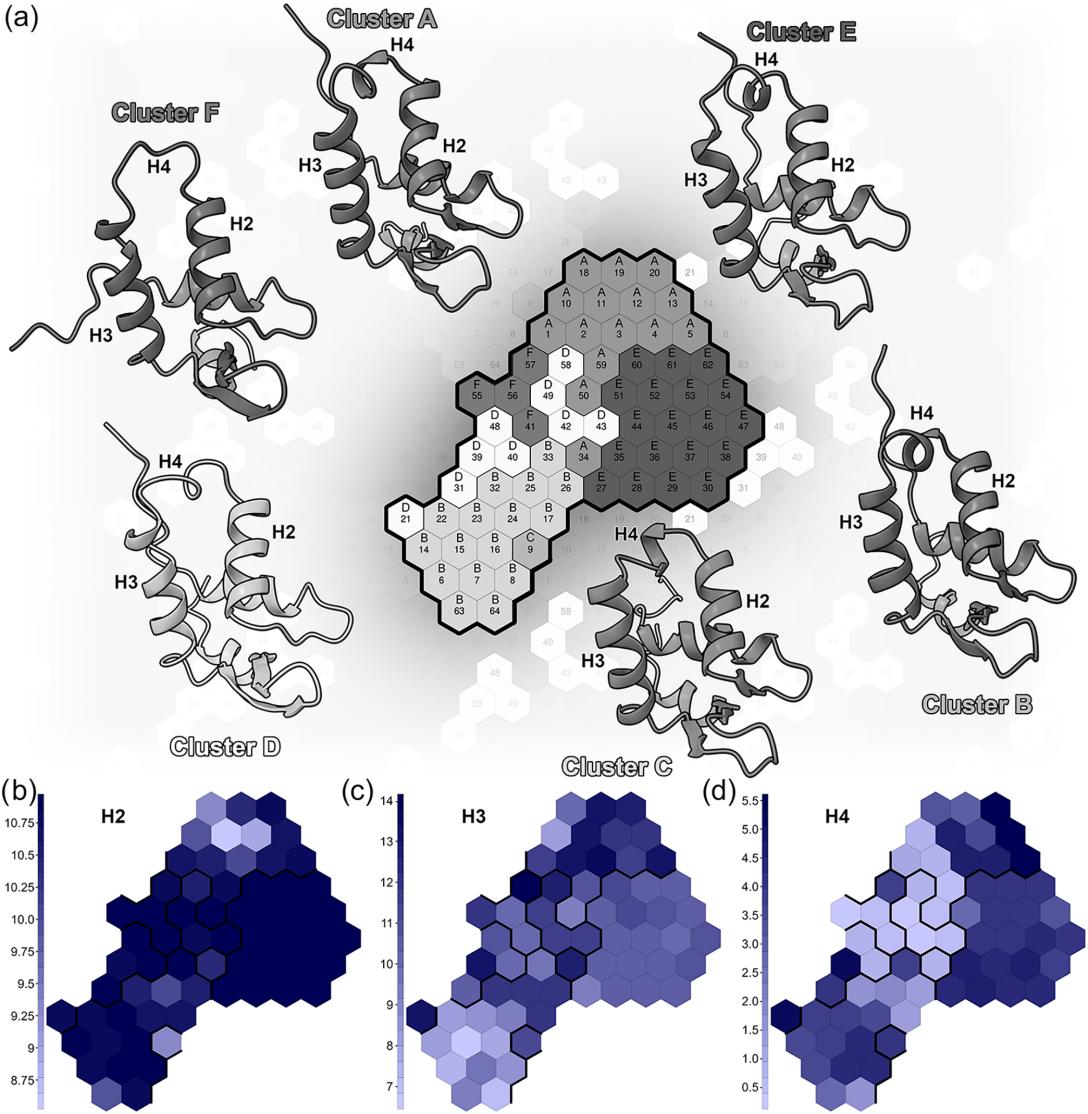
SOM clustering, representative structures, and regional helical propensity of the FOXP1 variants. (a) SOM clustering of the WT and FOXP1 variant simulations. The periodic (toroidal) nature of the map is highlighted by using transparency around the central boundaries to indicate continuity. The representative centroid conformation for each of the six identified macrostates is depicted as a cartoon, color-coded in shades of gray corresponding to the cluster assignments on the map. (b, d) Average helical content of the three central helices mapped across the SOM. Neurons are color-coded according to the average number of residues adopting an α-helical conformation in the H2 (b), H3 (c), and H4 (d) helices, computed from all MD frames assigned to that specific neuron. Darker blue shades indicate higher secondary structure persistence, whereas lighter shades reflect a reduced helical propensity or local melting. This combined representation allows for a direct correlation between the global 3D macrostates and the quantitative variations in the local secondary structure.

In line with our prior RMSF data (Figure 4), the region exhibiting the most substantial variations encompasses helices H2–H3–H4. The identified clusters effectively represent the structural changes occurring during the dynamics. While standard global RMSD reduces the structural deviation of the entire domain to a single scalar value, often masking localized but functionally critical fluctuations, our SOM framework separates the conformational ensemble based on the high-dimensional variance of the specific Cβ-Cβ distance network. By mapping the secondary structure persistence, computed via the DSSP algorithm, directly onto the SOM neurons (Figure 5b–d), we can explicitly identify the structural features driving this separation. Specifically, the SOM clustering perfectly captures and segregates the macrostates based on localized dynamics, such as the partial unfolding of the H3 recognition helix, the structural variability of the H4 region, and the relative reorientation of the H2 helix. Notably, the short additional H4 helix, situated between the H2 and H3 helices, displays significant variability, forming or disappearing during the simulations. Concurrently, the adjacent H2 and H3 helices may undergo changes in their secondary structure, resulting in more extended or contracted helices and, in some instances, even a break in the helical conformation.

To inspect how the mutations alter the dynamics of the system, we compared the percentages of frames falling within each cluster for each mutant (Figure 6). We found that the WT protein remains very close to the starting conformation, and samples only neurons (>99% of the frames coming from the 10 replicas) belonging to cluster E. This cluster can thus be assumed as the one representing the reference conformation, and indeed the experimental conformation also fall within this cluster.

**Figure 6:**
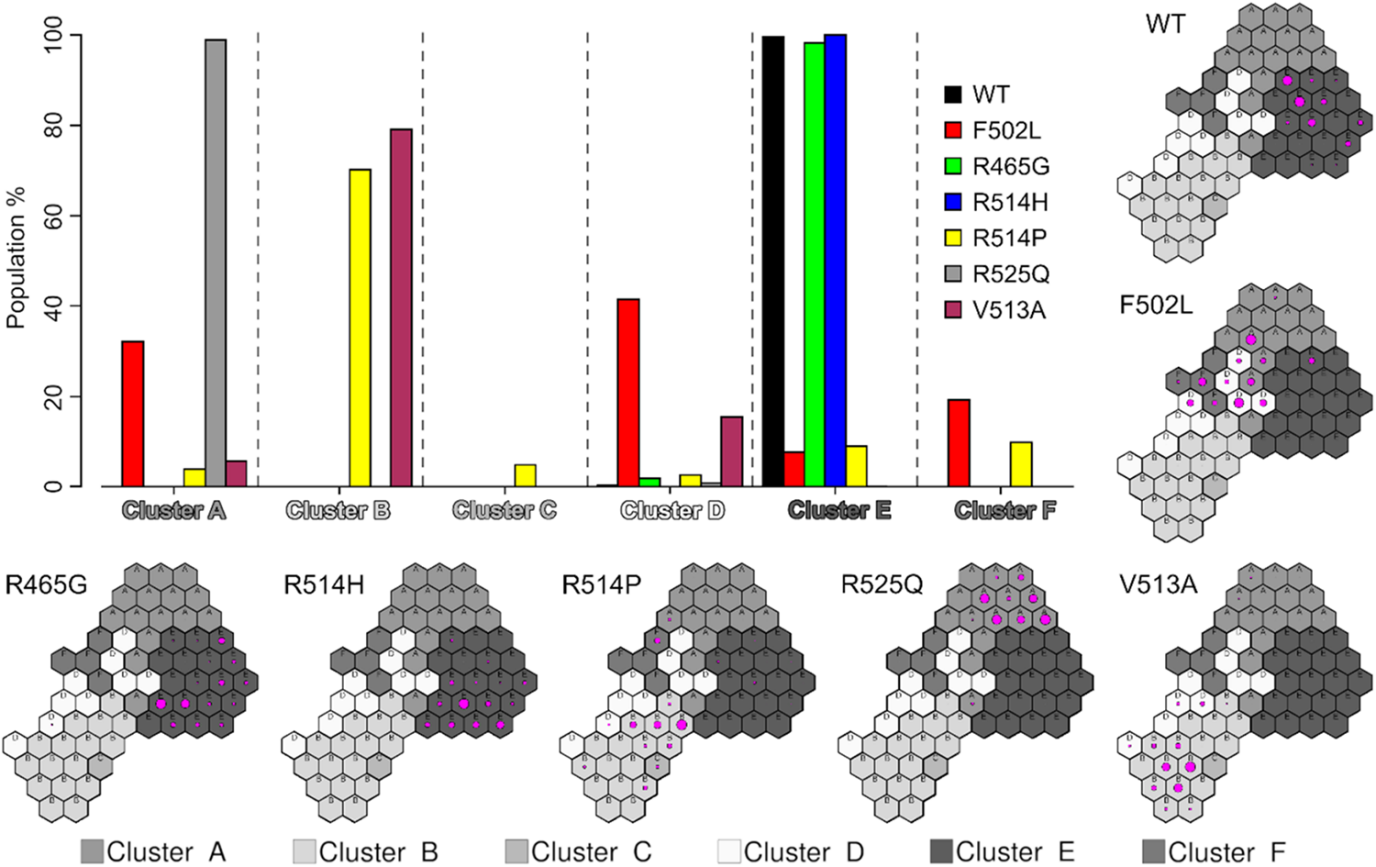
Percentage of MD frames falling within each cluster for the WT FOXP1 FKH domain and the six mutants analyzed in this work. The SOM population maps for each system are also reported, in which the size of the magenta circles is proportional to the number of frames of the neuron.

Mutants R465G and R514H display behavior comparable to that of the WT protein, with almost all their frames falling within cluster E. This is compatible with the observation that R465 falls within the N-terminal end of the domain and does not take part in the interactions that form the core of the domain. R514, on the other hand, is located within the H3 helix. In the experimental structure, the side chain of the arginine residue is completely exposed to the solvent, and thus, the mutation to another polar/charged residue is not expected to produce adverse effects on the overall structural integrity of the domain.

F502L, R514P, R525Q, and V513A, on the contrary, drift significantly from the starting conformation. While for most mutants it is possible to identify a predominant cluster, the F502L mutant samples three clusters (A, D, and F) with similar percentages (30, 40, and 20%, respectively). This can be interpreted as an indicator of the higher mobility of the F502L mutant, which indeed was the only mutant that displayed significantly higher RMSD values during the simulations (Figure 3). R514P and V513A mutants spend most of the simulated time within cluster B, while the R525Q mutant samples mostly cluster A. It is noteworthy that R514 and V513 are consecutive residues and fall within helix H3. Further, while the R514H mutant did not display altered conformations because the R514 side chain is completely exposed (see above), the R514P mutation had a different effect, since the substitution with a proline impacts the backbone of the residue, directly affecting the fold of helix H3. A similar effect is plausibly produced by the mutation of the adjacent residue, V513A, which might alter the helical propensity of helix H3.

### Mutations Impact Helix Propensity and DNA-Binding Competence

To understand the molecular basis for these altered dynamics, we investigated their impact on key structural features, starting with the secondary structure persistence of the H2, H3, and H4 helices. We computed the total number of residues adopting an α-helical structure in each frame of the simulation and presented the per-neuron average values on the map (Figures 5b–d and S1). We found that within the region of Cluster E (representing the WT-like conformation), all three helices exhibit consistent behavior, as indicated by similar values for the number of folded residues. In contrast, the other clusters display substantial variations in helix propensity. This map, along with the per-neuron populations for different mutants (Figure 6), provides insights into the local effects of mutations on the secondary structure persistence of FOXP1. Given that the observed changes in H2 length were small (≤2 residues), we interpreted these results as modest but reproducible shifts in the helix propensity.

For instance, the F502L mutant explores regions of the SOM map where almost all of the H4 residues remain unfolded. Given that residue F502 is located on H4, this mutation likely disrupts the local network of interactions, affecting the stability of this region. This residue is in fact situated within an aromatic environment, surrounded by residues such as Y470, Y492, F495, F499, Y501, and W509 WT (Figure 7), and contributes to form a crucial aromatic core essential for the proper folding of the FOXP1-DNA binding domain. Altering this residue affects the interaction network in the core of the protein, impairing the overall fold of the domain. More generally, the structural cues observed for F502 suggest a practical strategy to prioritize additional residues that may be sensitive to missense variation. Positions that are (i) buried within the FKH core, (ii) engage in persistent hydrophobic/aromatic contacts across the WT ensemble, and (iii) are highly connected within the intradomain contact network are likely to contribute disproportionately to fold integrity. In FOXP1, this criterion highlights several neighboring aromatic residues (Y470, Y492, F495, F499, Y501, and W509) as candidate structural “hotspots” whose substitution could similarly disrupt the aromatic packing network and alter the conformational ensemble. Future work will be needed to systematically test such predictions.

**Figure 7:**
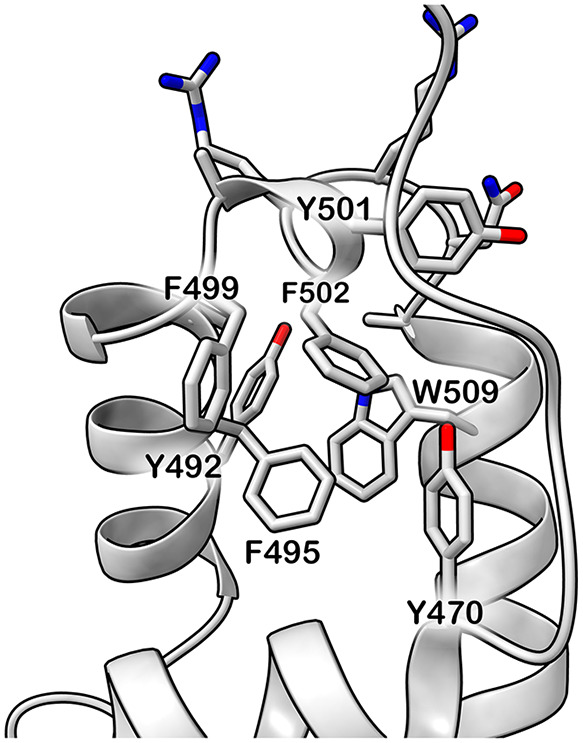
Structure of the FOXP1-DNA binding domain, with a focus on F502 residue. F502 and residues within 5 Å are displayed in sticks and aromatic residues are labeled.

Other mutations affect the persistence of the H3 helix. Interestingly, conformations in the bottom-left region of the SOM (predominantly cluster B) show a marked reduction in H3 α-helical propensity, with on average ∼7 of the 14 residues classified as α-helix. This pattern is consistent with local helix melting (often at the termini or short segments) rather than a single, large-scale unfolding transition. The V513A mutant explores neurons at the bottom of the map, leading to a lack of persistence in the H3 helix (residues 516–520). Similarly, the R514P mutation induces a break in the H3 helix, associated with the folding into an α-helix of the residues preceding H3 (residues 505–510) which are not folded in the WT (see also Supporting Figures 1 and 2). Since both residues are located within the H3 helix, they directly contribute to the induced changes. Lastly, the R525Q mutation appears to induce an extra fold in the region preceding helix H3 without causing a break in the middle region. This residue is distant from the region with conformational changes, suggesting that the extra stabilization effect likely propagates through the domain. Because DSSP-based helix assignment captures local hydrogen-bonding, whereas RMSF reflects positional fluctuations after global alignment, a decrease in helix propensity does not necessarily coincide with an increase in RMSF or an obvious “unfolding event” in the RMSD-based cluster descriptors.

Using the centroids of each neuron cluster obtained with the SOMs, we performed molecular docking simulations between the FKH domain of FOXP1 and its target dsDNA. Our docking analysis was guided by the crystal structure of the FOXP2-DNA complex, leveraging the conservation of DNA-binding residues between FOXP2 and FOXP1.^34,65^ Following the final refinement, each docking run yielded 200 structures of the FOXP1 FKH domain in complex with dsDNA, providing robust results. We utilized HADDOCK3 software, enabling CAPRI assessment of all 200 structures generated for each of the 64 neurons using the FOXP2-DNA crystal structure as the reference. The docking models were ranked based on the DockQ score. To visualize and interpret the docking results, we mapped the DockQ scores on the SOM (Figure 8). The DockQ score is a metric used to evaluate how closely a predicted docking pose (e.g., protein–DNA complex) matches a reference structure. Higher DockQ scores indicate a closer resemblance to the known structure. In our study, we found that conformations falling within cluster E (that includes mostly frames from the WT, R465G, and R514H simulations) generate binding poses that closely resemble those observed in the crystallographic structure (Figure 8).

**Figure 8:**
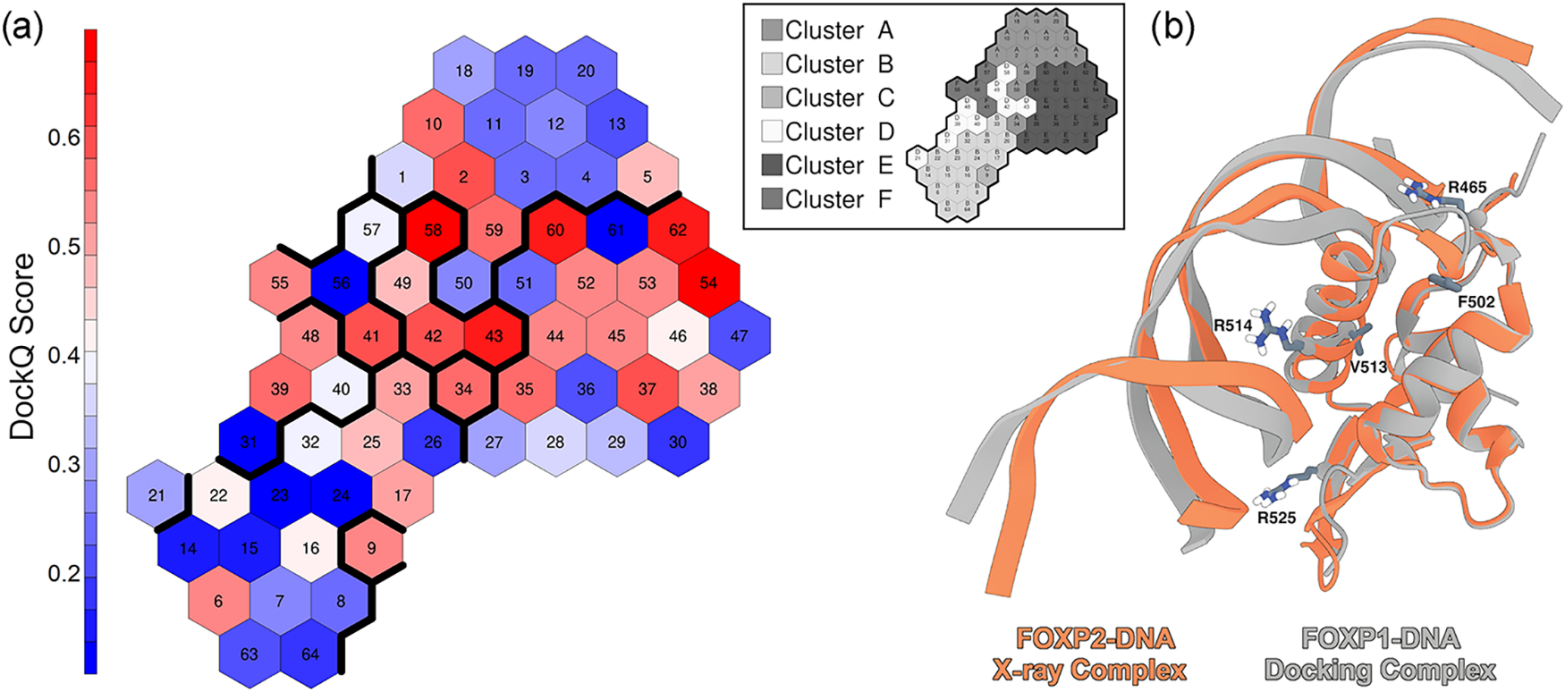
Average DockQ score mapped on the SOM. (a) Representation of DockQ scores mapped on the trained SOM. The color scale ranges from red to blue, with red values indicating more favorable scores. (b) Representation of the docking pose with the best DockQ score for the WT compared with the X-ray structure (PDB ID: 2A07).

To further validate the robustness of our docking predictions, we also mapped the native HADDOCK scores across the SOM (Supporting Figure 6). The topological distribution of the HADDOCK scores is consistent with DockQ mapping, confirming that the identification of the high-affinity macrostates is not biased by the scoring metric employed.

Only conformations belonging to neurons on the lower part of the cluster (neurons 26–30 and neuron 36) consistently displayed lower affinity. Interestingly, these neurons are mainly populated by frames from the R514H simulations (Figure 6), suggesting that this mutation may have an effect on DNA binding affinity. On the other hand, clusters A and B display conformations that do not appear to be favorable for DNA binding. These clusters contain frames from simulations of R514P, V513A, and R525Q. Finally, conformations belonging to clusters D and F display DockQ scores comparable to those obtained by cluster A. These clusters mainly contain frames from the F502L mutant simulation. This may suggest that the F502L mutant perturbs the native-state conformational ensemble of the domain, without producing a marked change in docking performance against the reference dsDNA sequence used in this study. To further validate our docking predictions and assess the structural persistence of the predicted protein–DNA interfaces, we performed explicit all-atom MD simulations of the WT, F502L, and R514P complexes (five independent 250 ns replicas per system). The time evolution of the intermolecular native contacts and the complex backbone RMSD confirmed the docking-based trends (Supporting Figure 7). Specifically, the R514P complex exhibited a marked decrease in the number of native contacts and the highest conformational drift, indicating a clear disruption of the DNA-binding mode. In contrast, the F502L mutant maintained an interfacial native contact profile and a structural persistence that closely resembled those of the WT. This evidence corroborates our structural hypothesis: while the F502L mutation induces an internal perturbation within the hydrophobic core of the FKH domain, this internal rearrangement does not severely compromise the geometry or persistence of the DNA-binding interface.

## Discussion

Interpreting the pathogenicity of missense variants emerging from sequencing of clinical populations represents a significant and urgent challenge. Only ∼2% of the missense variants observed in the human genome are estimated to be unambiguously classified as clinically pathogenic or benign.^6^ Incorporating the analysis of 3D structural information predicted via MD simulations and machine learning has led to improvements in variant interpretation.^6^

In this study, we demonstrated the power of an integrated approach that combines MD simulations with an unsupervised machine learning technique, SOMs, to dissect the molecular consequences of pathogenic variants associated with FOXP1 syndrome. This strategy allowed us to move beyond global protein stability metrics and create a detailed map of the conformational macrostates populated by the wild-type and mutant proteins, providing a richer understanding of their behavior. A key strength of our methodological pipeline is its ability to reveal dynamic perturbations that are not immediately evident from standard analyses like RMSD and RMSF. Among the six missense variants falling in the FKH DNA-binding domain in our analyses, F502L revealed the most significant perturbation, detected by both RMSD and RMSF and further analyses with SOM. The impact of F502L is primarily due to the disruption of favorable interactions with the neighboring aromatic side chains to form a hydrophobic core within the protein domain. Surprisingly, despite this significant perturbation of the hydrophobic core, our docking predictions and short-time-scale MD simulations of the complex did not show an immediate abrogation of DNA binding. This apparent WT-like interface persistence is likely due to the limited time scale of the simulations, during which the global backbone has not yet fully collapsed. However, the continuous structural drift observed in the mutant trajectories likely represents the early stages of larger-scale destabilization. We speculate that, over longer biological time scales, this core instability will ultimately compromise the global architecture of the FKH domain, leading to impaired DNA recognition or premature protein degradation (turnover) *in vivo*. There is currently no experimental evidence of the functional consequences of F502L. Furthermore, despite no discernible alterations in mobility detected by RMSD and RMSF analyses, SOMs revealed an impact on protein conformational deviation, most notably affecting the folding of the H3 and H4 helices, for V513A, R514P, and R525Q. On the contrary, the R465G and R514H mutants explored a conformational space similar to that of the WT protein. Importantly, our simulations were performed on an isolated FKH domain construct, thus capturing the variant effects within this domain and the DNA recognition surface of the protein. This design enables a systematic variant-by-variant comparison of conformational macrostates. However, it does not explicitly represent full-length FOXP1, or higher-order assemblies/partner-dependent complexes that contribute to the FOXP1 function. Accordingly, the conclusions drawn here should be interpreted as domain-resolved mechanistic insights that motivate future modeling of multidomain and/or oligomeric states, rather than as a limitation of MD. Another limitation is that the mutant models were generated by *in silico* substitution of a folded wild-type structure. The simulations primarily probed how each variant perturbed the native-state conformational ensemble, local structural persistence, and DNA interaction competence rather than recapitulating *de novo* folding pathways. Standard MD, starting from a folded template, may not capture cases in which a variant substantially alters the folding kinetics, stabilizes an alternative fold, or promotes partial unfolding over longer time scales. Moreover, physiological ionic strength may influence absolute DNA-binding energetics. Therefore, future work will extend the simulations to include explicit salt concentrations (e.g., 150 mM NaCl) to quantify ionic strength sensitivity.

Mutations that directly affect the DNA-binding interface (V513A, R514H, R514P, and R525Q) displayed altered docking performances and reduced interface persistence. Interestingly, most of them perturbed the conformation of the H3 helix, decreasing the DockQ docking score. Furthermore, explicit MD simulations of the R514P complex confirmed a rapid loss of native intermolecular contacts, validating its disruptive effect on DNA engagement. However, the R514H mutation seems to maintain WT-like dynamics, though the altered physicochemical properties at the interface contribute to diminished DNA binding affinity. On the other hand, while V513A is not expected to significantly alter the composition of the protein-interacting surface, the altered conformation induced by this mutation appears inadequate for effective DNA binding.

The predictions generated by our computational model find strong support in the existing experimental data. The functional impact of FOXP1 missense variants has been experimentally tested by exogenously expressing mutants in non-neuronal cell lines.^15,41,43^ More recent evidence in human forebrain organoids carrying the heterozygous R514H mutation indicates a loss of heterodimerization with FOXP4 and ∼40% reduction in DNA-binding events, with alterations in the expression of genes necessary for neuronal development.^77^

In-depth structural information about the perturbations introduced by missense *FOXP1* variation can aid in the design of novel therapeutics aiming at stabilizing protein–protein or protein–DNA interactions. Further, in addition to the value of our analyses for understanding the impact of variation associated with FOXP1 syndrome, our approach combining MD and SOMs can be readily applied to the comparative analysis of the dynamics of other biological systems, offering valuable insights into their behavior and potential functional consequences.

In conclusion, this work presents a robust and generalizable computational pipeline for the mechanistic interpretation of missense variants. While providing critical insights into FOXP1 syndrome, the primary contribution of our study lies in demonstrating how the synergy between large-scale MD simulations and machine learning can effectively map complex conformational landscapes and link them to functional outcomes. This approach can be readily applied to the comparative analysis of other biological systems, where understanding the subtle impact of genetic variation remains a key challenge, offering a powerful strategy to bridge the gap between genotype and molecular phenotype.

## Supplementary Material



## Data Availability

The data underlying this article are available at https://data.mendeley.com/datasets/hn97ftwgx4/1 (doi:10.17632/hn97ftwgx4.1)

## Notes

Time-dependent secondary structure analysis of WT and mutant FOXP1 variants, Silhouette score profiles for SOM cluster optimization, structural analysis of protein–DNA docking and comparison with FOXP2, monitoring of internal protein contacts during simulations, and structural persistence of the FOXP1-DNA complexes during explicit solvent MD (Supporting Figures 1–7) (PDF)

## ⧓

Deceased

## Abbreviations

ASD: autism spectrum disorder
CAPRI: Critical Assessment of PRedicted Interactions
dsDNA: double-stranded DNA
FKH: forkhead
FOXP1: forkhead box protein P1
LINCS: Linear Constraint Solver
MD: molecular dynamics
NMR: nuclear magnetic resonance
NPT: constant number of particles, pressure, and temperature
NVT: constant number of particles, volume, and temperature
PDB: Protein Data Bank
RMSD: Root Mean Square Deviation
RMSF: Root Mean Square Fluctuation
SOM: Self-Organizing Map
WT: wild-type
ZIP: leucine zipper
