# Supplementary material for: Examining Genetic Variants Associated with FOXP1 Syndrome through Molecular Dynamics of Its DNA-Binding Domain and Self-Organizing Maps
Source: J Chem Inf Model. 2026 Apr 17;66(9):5078–90. doi: 10.1021/acs.jcim.6c00080 (PMC13169393; doi:10.1021/acs.jcim.6c00080)
Supplement: Supplementary file 1 [file ci6c00080_si_001.pdf]

Examining genetic variants associated with FOXP1 syndrome through molecular dynamics of its DNA-binding domain and self-organizing maps

Stefano Motta[1,2 †*], *Nunzio Perta*[3,4,5†], *Alice Romagnoli*[3,4,5], *Jesmina Rexha*[3,4,5], *Joseph D Buxbaum*[6,7,8,9], *Silvia De Rubeis*[6,7,8,9,10 *], *Daniele Di Marino*[3,4,5, #]

[1] Department of Earth and Environmental Sciences, University of Milano-Bicocca, Milano, MI, 20126, Italy.

[2] Inter-University Center for the Promotion of the 3Rs Principles in Teaching & Research (Centro 3R), Pisa, 56122, Italy.

[3] Department of Life and Environmental Sciences, Poly

technic University of Marche, Ancona, AN, 60131, Italy.

[4] New York-Marche Structural Biology Center, Polytechnic University of Marche, Ancona, AN, 60131, Italy.

[5] Department of Neuroscience, Mario Negri Institute for Pharmacological Research-IRCCS, Milano, MI, 20156, Italy.

[6] Seaver Autism Center for Research and Treatment, Icahn School of Medicine at Mount Sinai, New York, NY, 10029, US.

[7] Department of Psychiatry, Icahn School of Medicine at Mount Sinai, New York, NY, 10029, US.

[8] The Mindich Child Health and Development Institute, Icahn School of Medicine at Mount Sinai, New York, NY, 10029, US.

[9] Friedman Brain Institute, Icahn School of Medicine at Mount Sinai, New York, NY, 10029, US.

[10] Alper Center for Neural Development and Regeneration Friedman Brain Institute, Icahn School of Medicine at Mount Sinai, New York, NY, 10029, US.

[†] Joint Authors

* To whom correspondence should be addressed. Stefano Motta, stefano.motta@unimib.it; Silvia De Rubeis, silvia.derubeis@mssm.edu

Deceased

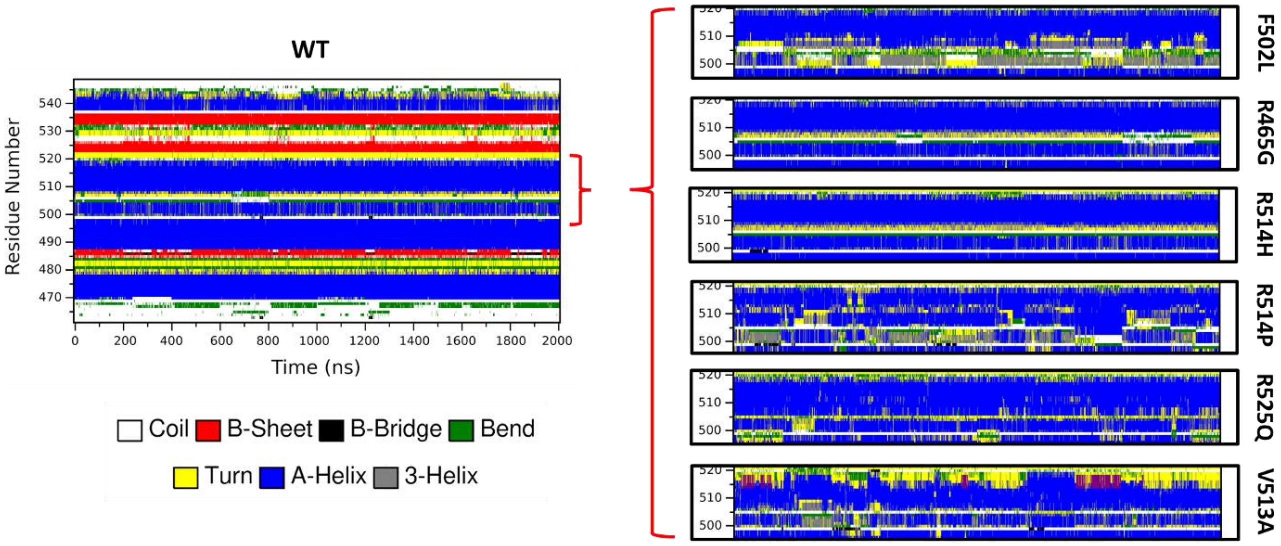

Supplementary Figure 1. Secondary structure analysis during MD simulations of the WT (left) and details for the H3 and H4 helices of the mutants (right).

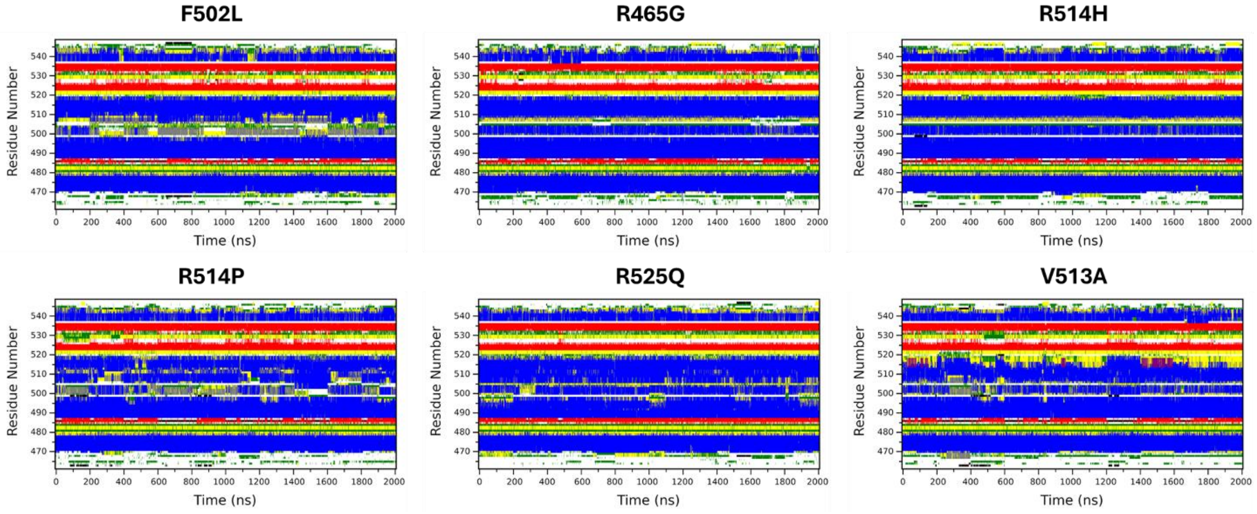

Supplementary Figure 2. Secondary structure analysis for the whole protein, during MD simulations for the variants object of the study.

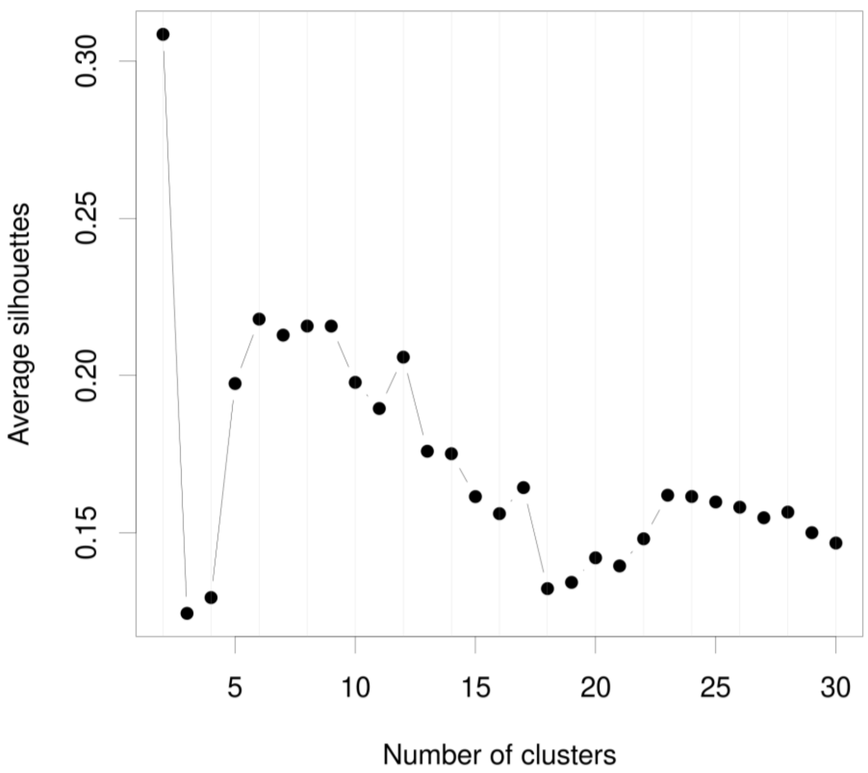

Supplementary Figure 3. Silhouette score profile for the neuron-clustering phase. The optimal number of clusters (6) was chosen as the first maximum in the profile.

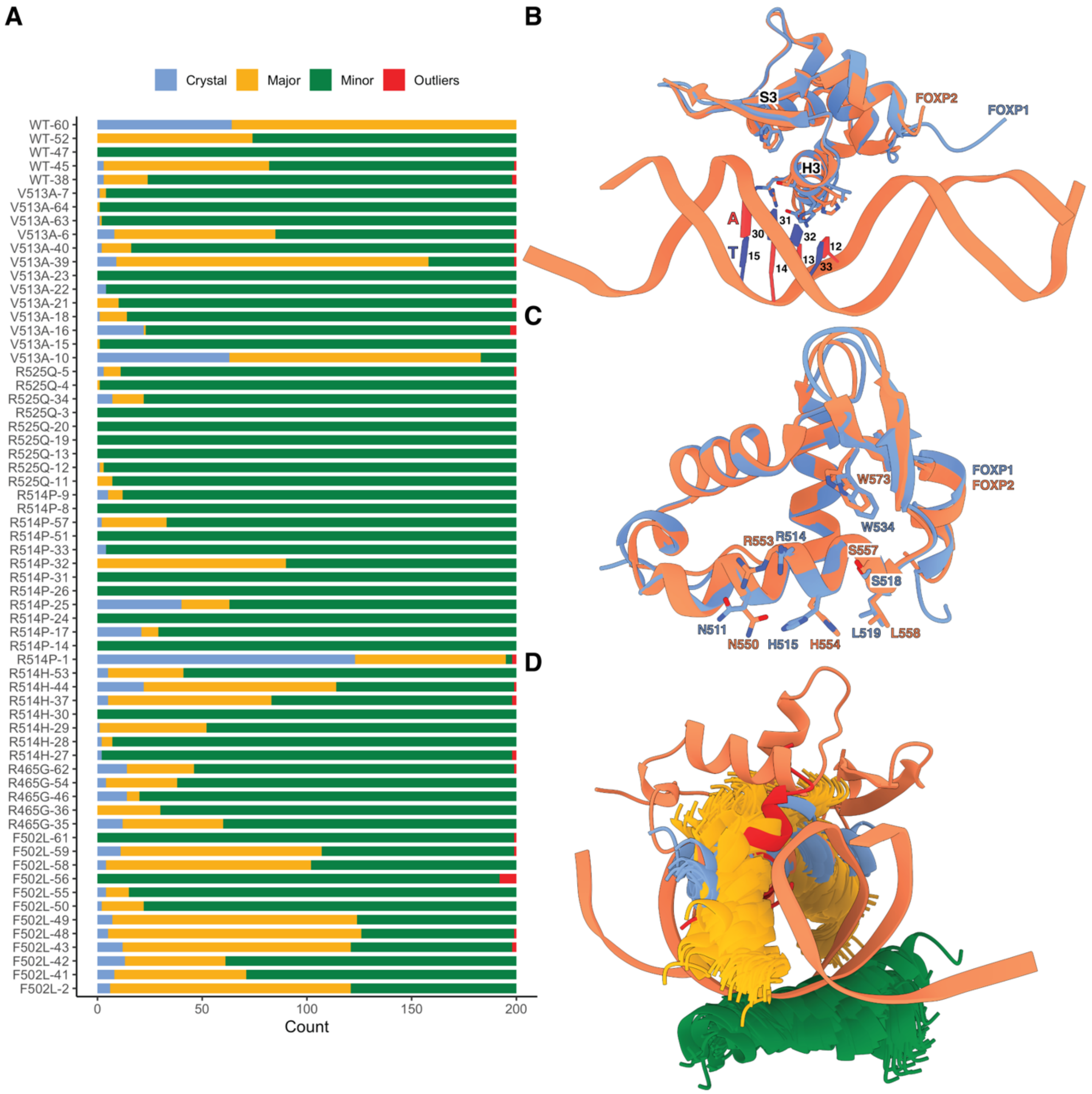

Supplementary Figure 4. Structural analysis and docking results of FOXP1 and FOXP2 forkhead (FKH) domain with DNA binding. A) Bar plot illustrating the number of distinct poses obtained for each neuron, grouped by wild-type (WT) and mutants according to the Self-Organizing Map (SOM). Stacked poses are color-coded as per the legend. **B**) Ribbon representation of the FKH domain superimposition of FOXP1 and FOXP2 proteins bound to double-stranded DNA (dsDNA) (strand 1-21: 5'-AACTATGAAACAAATTTTCCT-3' and strand 22-42: 5'-TTAGGAAAATTTGTTTCATAG-3'). The DNA nucleotides used as active residues in docking are highlighted on the ribbon representation of DNA, with adenine and thymine depicted in coral and purple, respectively. **C**) Ribbon representation of the FKH domain superimposition of FOXP1 and FOXP2 proteins (RMSD between pruned atom pairs is 1.2 Å). Conserved residues used as actives for FOXP1 during docking are shown as sticks and labeled with structure-specific colors (light salmon for FOXP2 and cornflower blue for FOXP1). **D**) Ribbon depiction of a representative docking result for a F502L neuron (number 43). Each complex is aligned based on dsDNA from the target complex of FOXP2-DNA and shows only the H3 helix of each FOXP1 FKH domain structure (residues 506-520). Same color coding as in A.

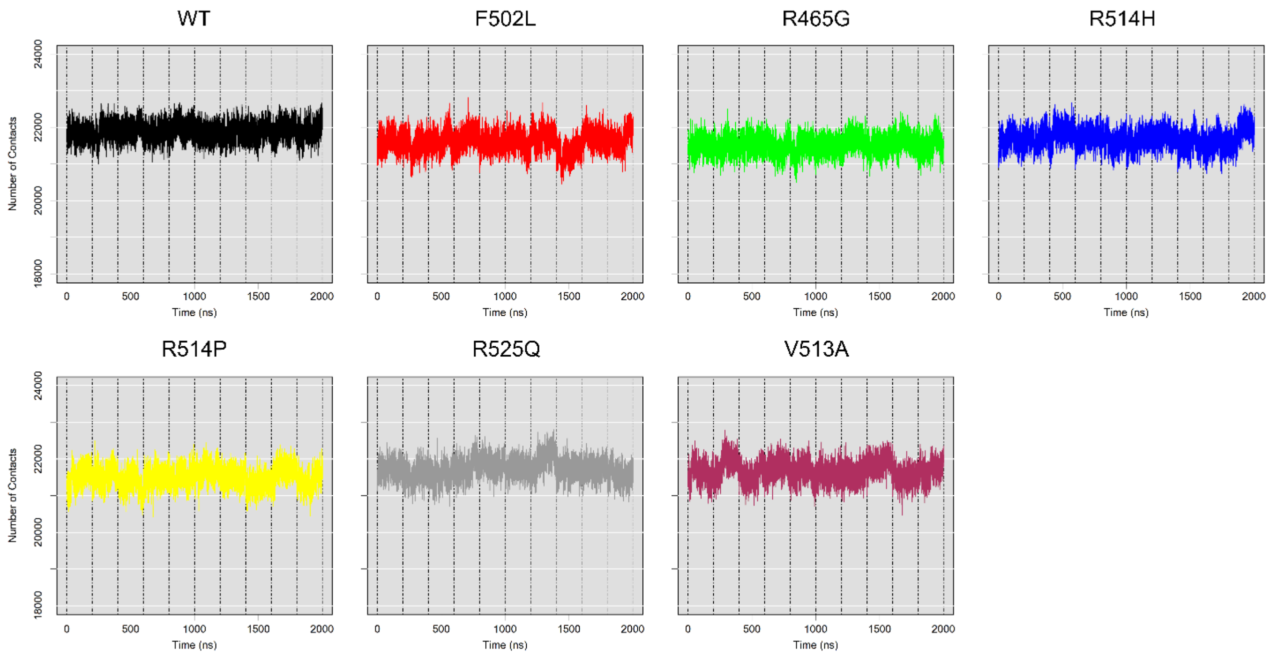

Supplementary Figure 5. Monitor of the number of internal contacts for conformations sampled during the concatenated MD replicas of FOXP1 WT and mutated systems.

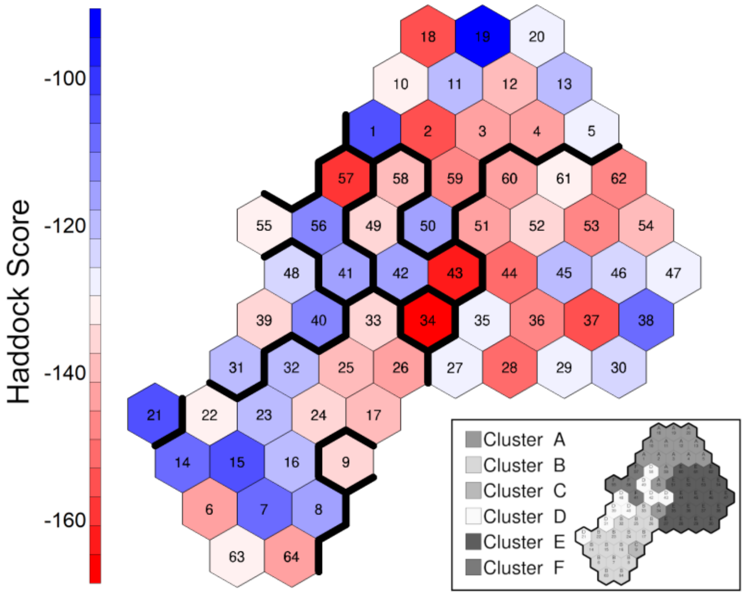

Supplementary Figure 6. Average HADDOCK score mapped on the SOM. Representation of the HADDOCK scores calculated for the representative docking poses and mapped onto the trained SOM. The color scale ranges from red to blue, with red values indicating more favorable (more negative) energetic scores.

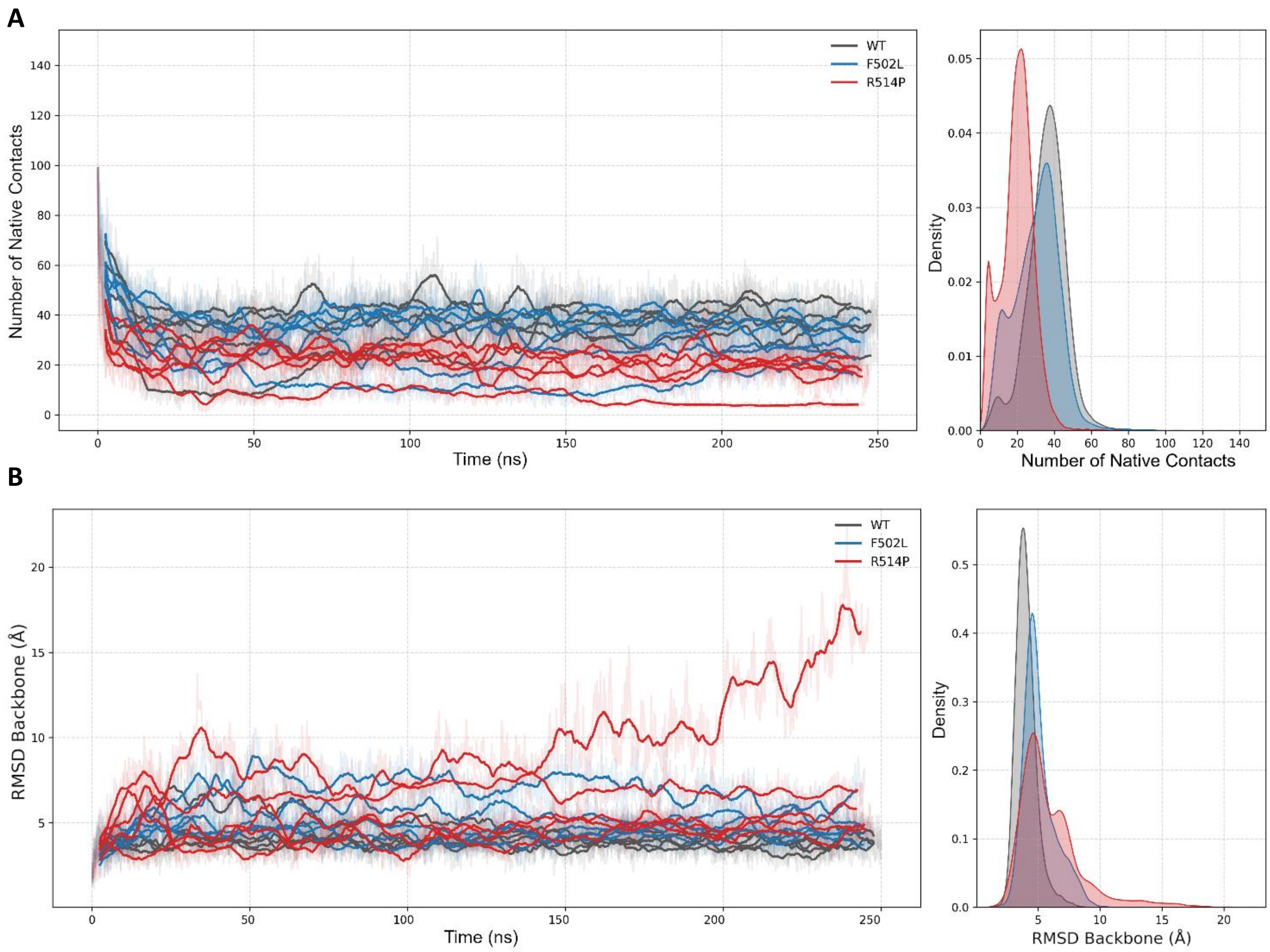

Supplementary Figure 7. Structural analysis of the FOXP1-DNA complexes during explicit solvent molecular dynamics. A) Time evolution (left) and corresponding probability density (right) of the number of native contacts at the protein-DNA interface. Native contacts were computed between protein heavy atoms and DNA heavy atoms using a distance cutoff of 4.0 Å. Data are shown for the WT (grey), F502L (blue), and R514P (red) complexes across five independent 250-ns replicas per system. Solid lines represent smoothed averages, while lighter transparent lines show the raw fluctuations. **B**) Time evolution (left) and probability density (right) of the protein backbone Root Mean Square Deviation (RMSD). The RMSD was calculated on the protein backbone atoms relative to the initial docked reference conformation, following structural alignment of the trajectories on the DNA phosphorus atoms.
